# Local dissemination of osteosarcoma observed after massage therapy: a case report

**DOI:** 10.1186/s12885-019-6246-4

**Published:** 2019-10-23

**Authors:** Shinji Miwa, Michi Kamei, Satoru Yoshida, Satoshi Yamada, Hisaki Aiba, Hiroyuki Tsuchiya, Takanobu Otsuka

**Affiliations:** 1 Department of Orthopedic Surgery, Graduate School of Medical Science, Nagoya City University, Nagoya, Japan; 2 Department of Orthopedic Surgery, Graduate School of Medical Science, Kanazawa University, Kanazawa, Japan; 3 Department of Neonatology and Pediatrics, Graduate School of Medical Science, Nagoya City University, Nagoya, Japan

**Keywords:** Osteosarcoma, Dissemination, Massage therapy

## Abstract

**Background:**

Limited evidence is available regarding the dissemination of tumor tissues due to compression during massage therapy, a routine procedure in patients with various symptoms in Asian countries.

**Case presentation:**

A 12-year-old male presented at a massage clinic with pain and swelling of his left knee, which worsened the same night. Consistent with conventional osteosarcoma, radiography revealed cortical bone destruction, osteoblastic changes, and periosteal reactions. Magnetic resonance imaging revealed a tumor in the distal femur, an extraskeletal mass, and an infiltrative lesion in the intramuscular and neurovascular areas surrounding the distal femur; this was considered as hemorrhage and dissemination of the tumor tissue. ^18^Fluorine-labelled fluorodeoxyglucose-positron emission tomography and computed tomography revealed multiple metastases in the spine, liver, and lung. Consistent with osteosarcoma, histopathological examination revealed tumor cell proliferation with extensive pleomorphism and mitoses. Despite undergoing chemotherapy, radiation therapy, and hip disarticulation, the patient died due to multiple metastases 13 months after the initial diagnosis.

**Conclusions:**

The present case suggests association of massage therapy with the local dissemination of tumor tissues, although influence of massage therapy on metastatic lesions remains unclear. Massage therapists should be aware of the possibility for dissemination of hidden malignancies due to the procedure.

## Background

Despite it being the most common primary malignancy of the bone in adolescents and young adults, the incidence of osteosarcoma is only 5–7 cases/million/year [1]. Standard treatment modalities for osteosarcoma include preoperative chemotherapy, tumor resection with surgical margin, and postoperative chemotherapy. Prior to the introduction of chemotherapy, long term survival rates were < 20% [2, 3]; however, chemotherapy has significantly improved outcomes [4–6]. The current 5-year survival rate in patients with osteosarcoma is approximately 60–70% [7, 8]. Furthermore, limb-sparing surgery has become the standard surgical procedure since the introduction of chemotherapy, and 85–97% of patients with osteosarcoma have reportedly undergone limb-sparing surgery [9, 10].

Osteosarcoma most commonly affects the distal femur [11], and patients with osteosarcoma of the distal femur sometimes present with knee pain. The discrepancy between the lesion site and symptoms may lead to delayed diagnosis and inadequate treatments. Particularly in Asian countries, massage therapy is used for a variety of health-related purposes [12, 13]. Patients with malignancies sometimes receive massage therapy to alleviate symptoms including pain, swelling, and numbness. On the other hand, compression of tumor tissue may cause infiltration and metastasis although there is no clear evidence to support this process. Here we present a case suggesting the influence of compression of osteosarcoma on local dissemination of tumor tissue and discuss the effect of massage on the clinical course of tumor lesions.

## Case presentation

**Fig. 1:**
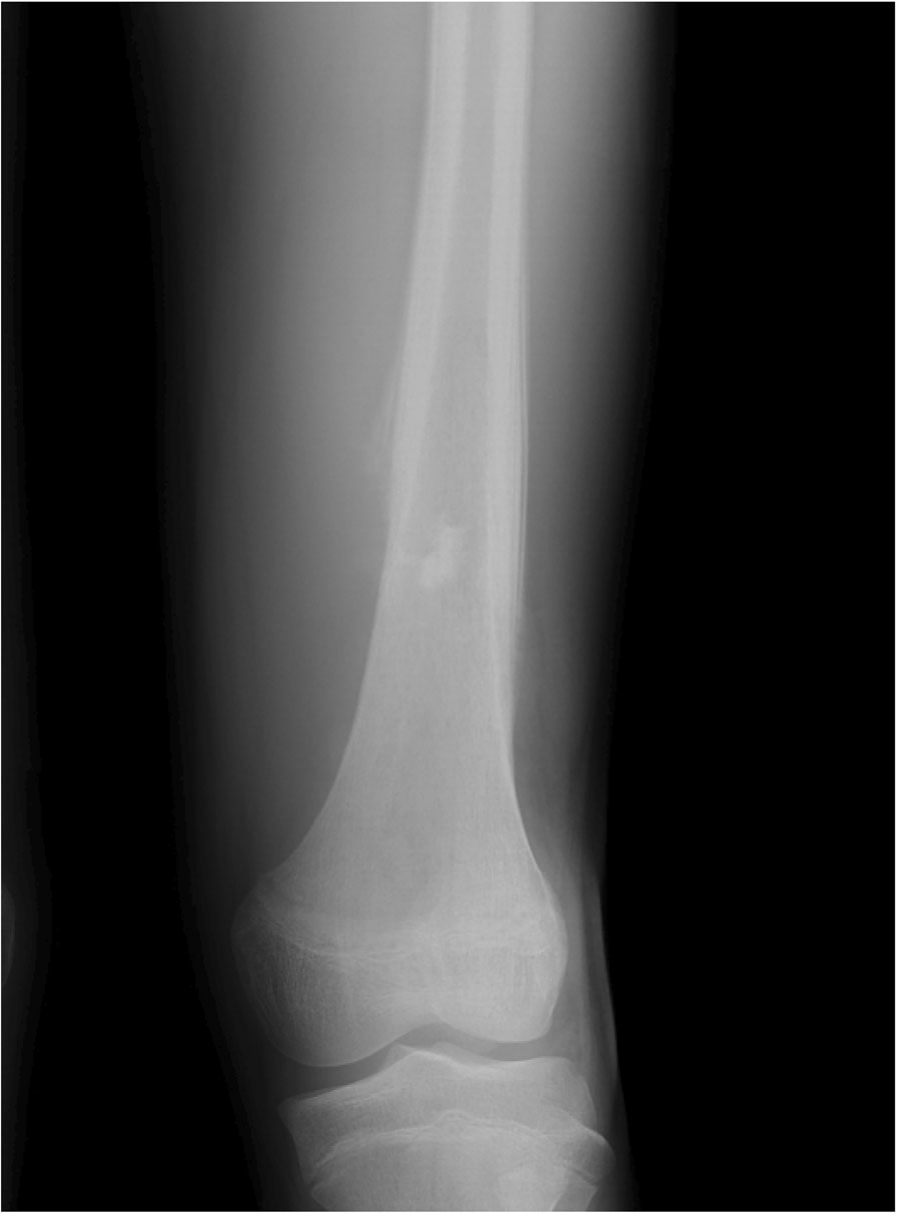
Radiograph before chemotherapy. Sclerotic lesion with periosteal reaction was observed in the distal femur

**Fig. 2:**
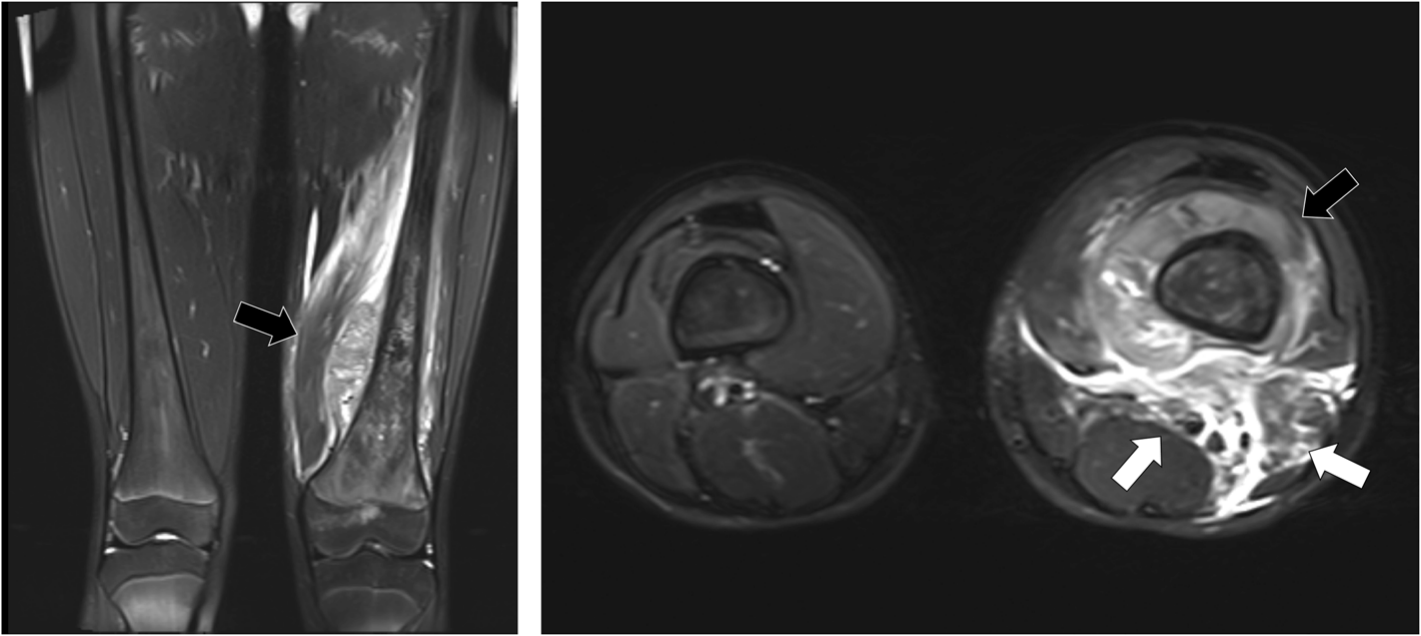
Magnetic resonance imaging (MRI) prior to chemotherapy. MRI revealed extraskeletal mass of distal femur (black arrow), and a lesion thought to be hemorrhage and dissemination of tumor tissues (arrow) were observed

**Fig. 3:**
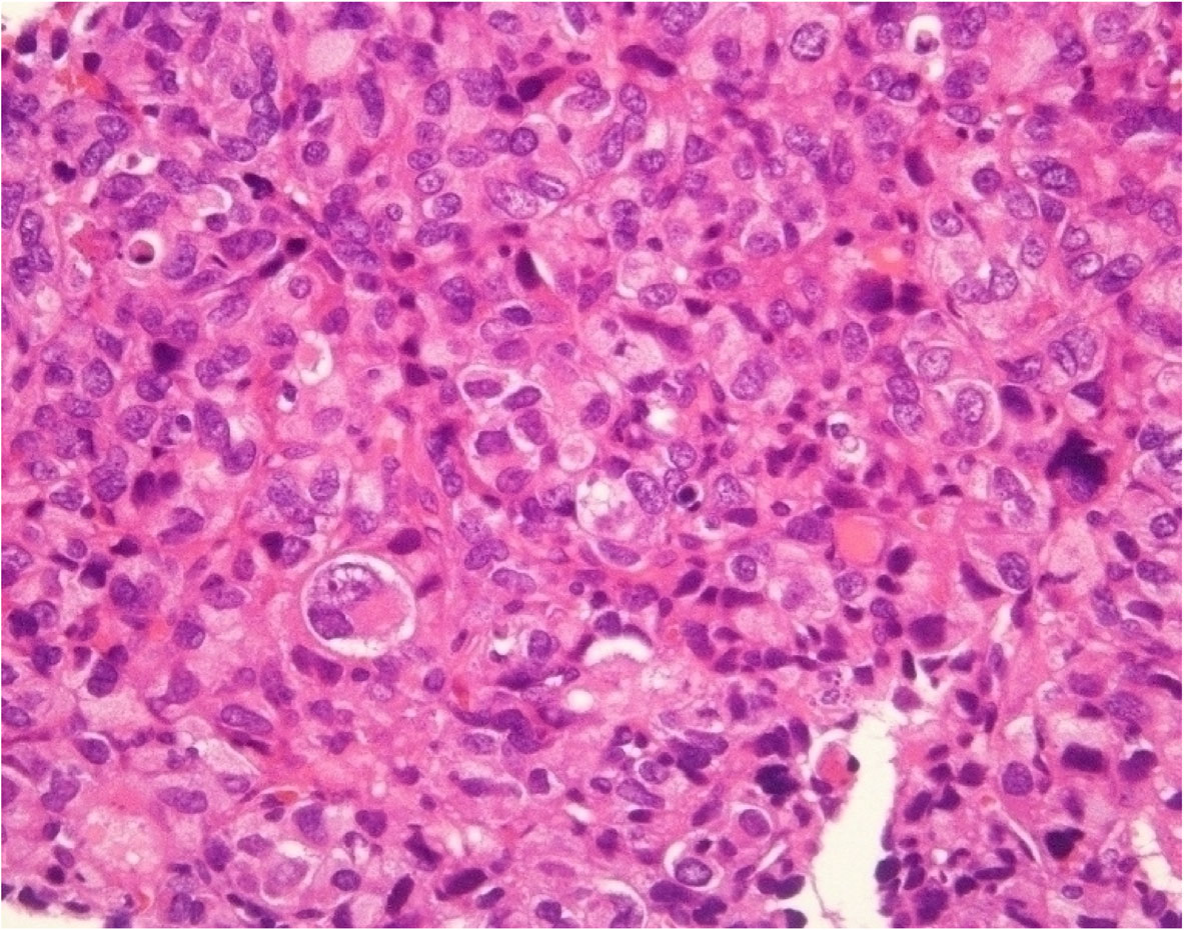
Histology. Hematoxylin and eosin staining showed proliferation of tumor cells with extensive pleomorphism and mitoses, which was consistent with osteosarcoma

**Fig. 4:**
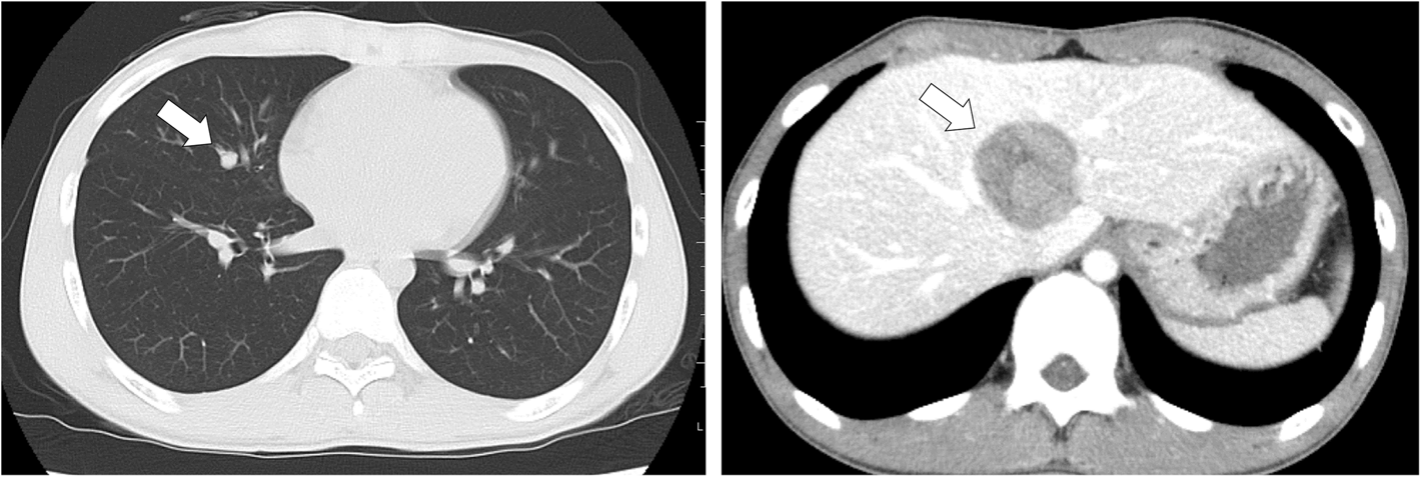
Computed tomography (CT) prior to chemotherapy. Metastatic lesions in the lung and liver were observed (arrow)

**Fig. 5:**
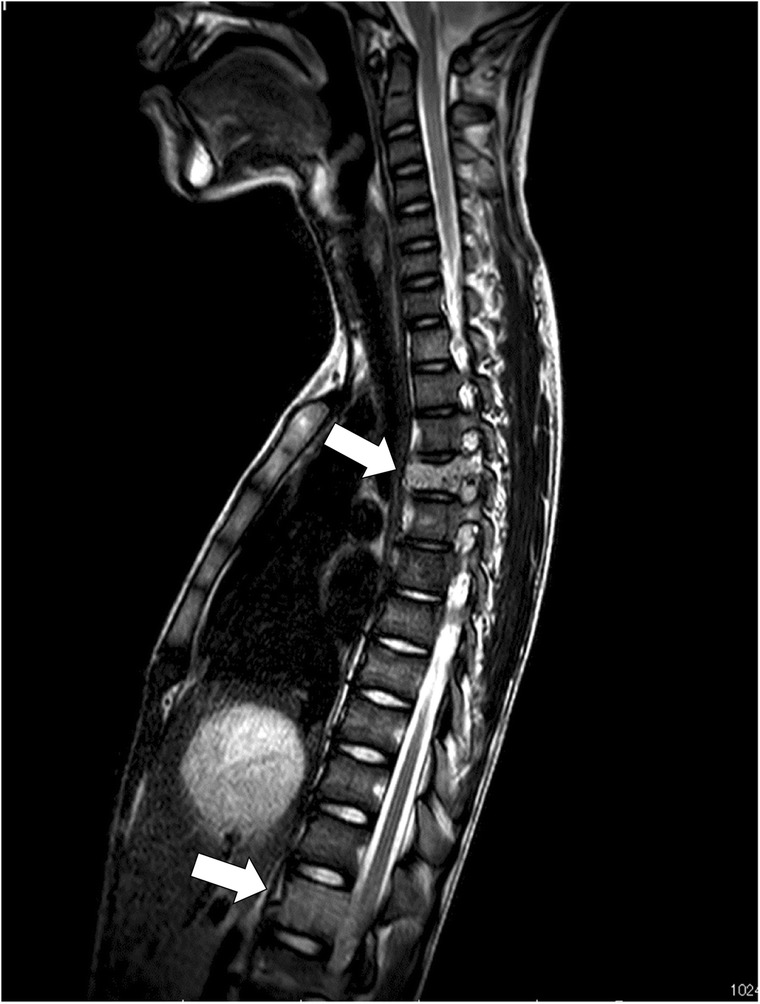
Magnetic resonance imaging. Metastases were observed at the Th4 and Th 12 vertebrae (arrow)

**Fig. 6:**
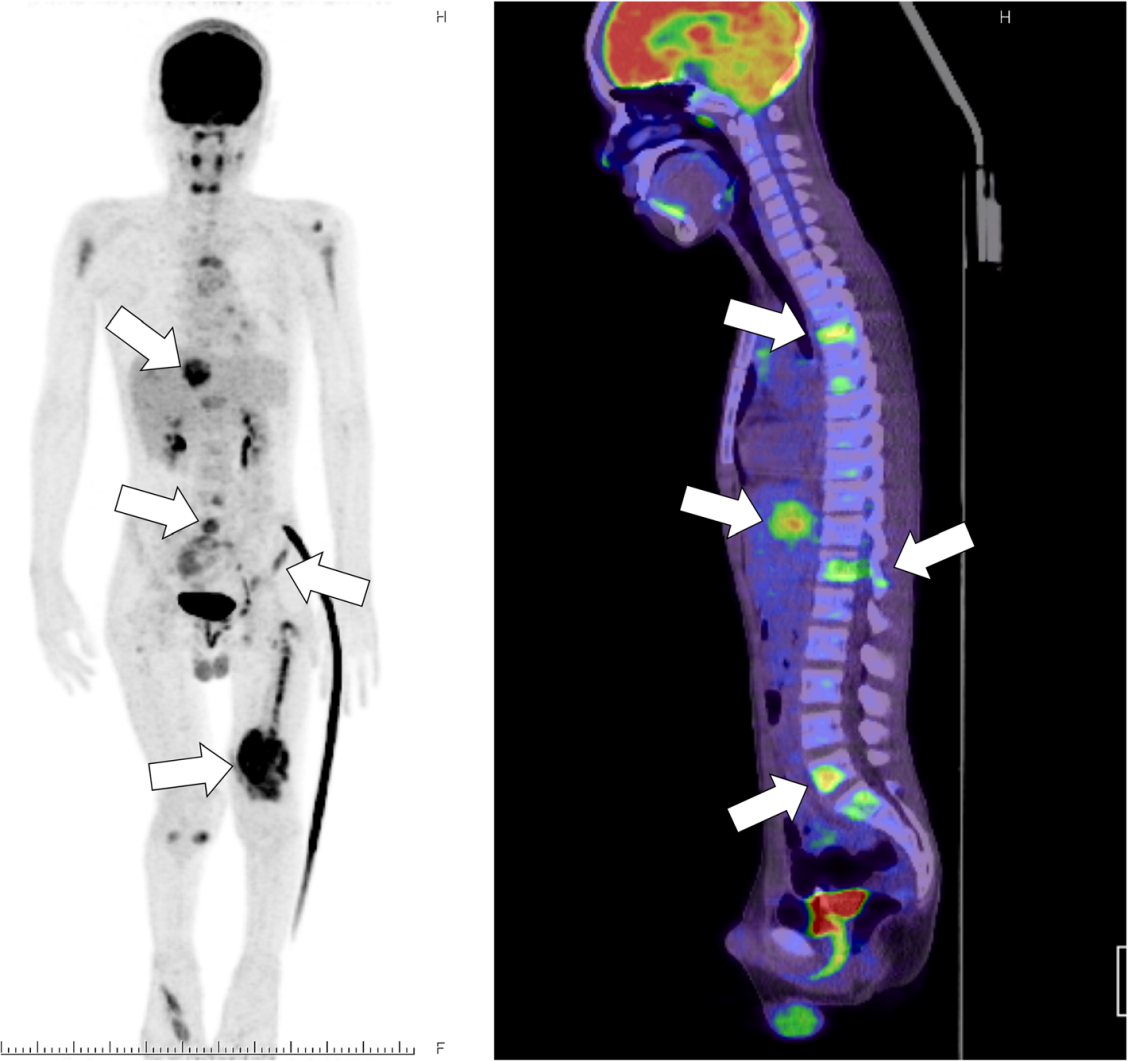
^18^Fluorine-labeled fluorodeoxyglucose–positron emission tomography. Multiple metastatic lesions were observed in the liver, spine, and pelvis

**Fig. 7:**
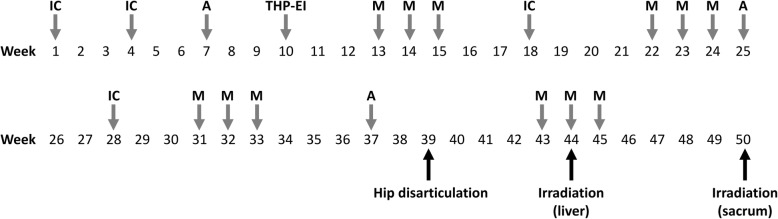
Treatment courses. IC: ifosfamide (2.65 g/m^2^ daily for 3 days) and carboplatin (560 mg/m^2^ on Day 1); THP-EI: Pirarubicin (50 mg/m^2^ on Day 1), etoposide (125 mg/m^2^ at Day 1 and Day 4), and ifosfamide (1500 mg/m^2^ daily for 4 days); A: doxorubicin (25 mg/m^2^ daily for 3 days); M: methotrexate (12 g/m^2^); A: doxorubicin (30 mg/m^2^ daily for 3 days)

**Fig. 8:**
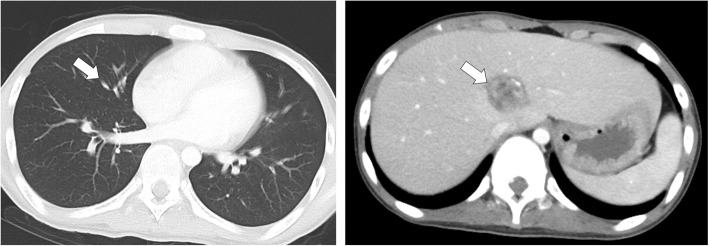
Computed tomography (CT) after chemotherapy. Reductions in the tumor volumes of metastatic lesions were observed in the lung and liver

**Fig. 9:**
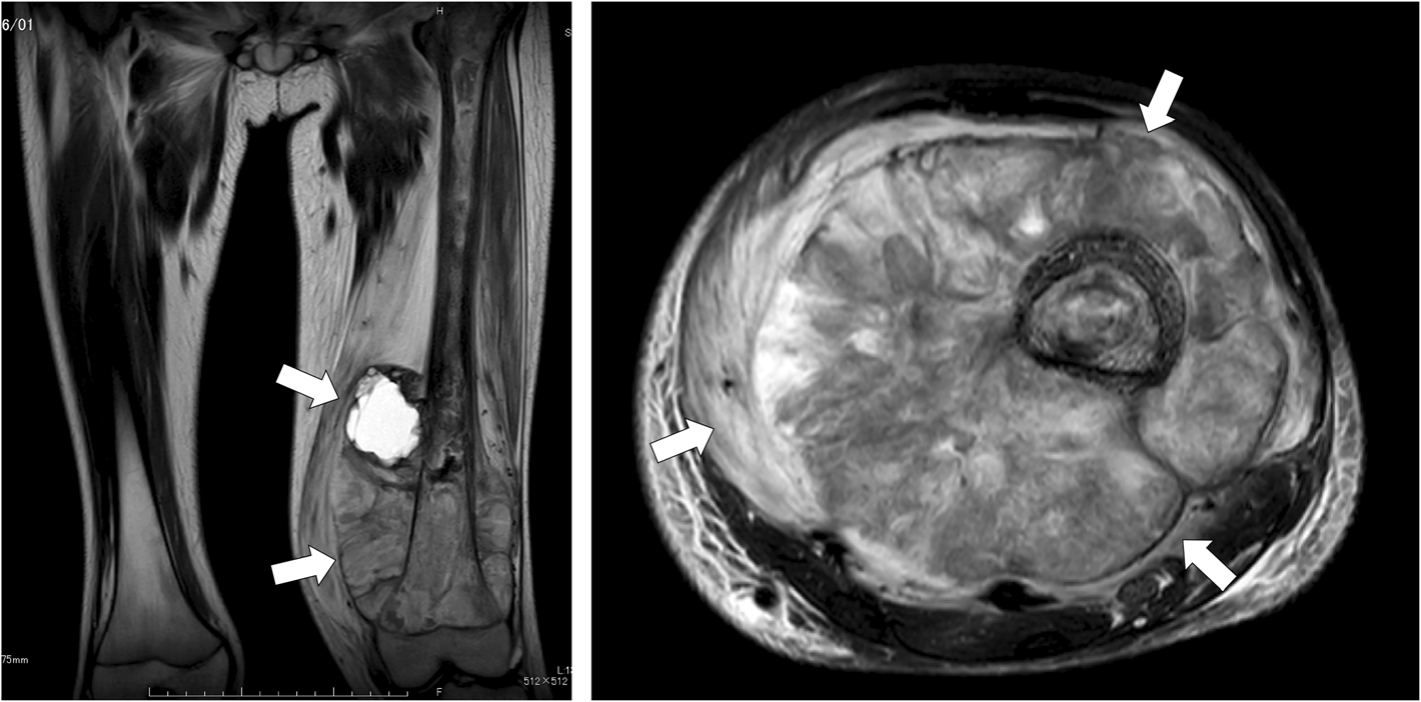
Magnetic resonance imaging after chemotherapy. Significant increase in the tumor size was observed in the distal femur

A 12-year-old male presented at a massage clinic with pain and swelling in his left knee, which worsened the same night. At the orthopedic clinic, radiography performed on the following day revealed cortical bone destruction, osteoblastic changes, and periosteal reactions, consistent with conventional osteosarcoma (Fig. 1). For further examination and treatment, the patient was referred to our hospital 5 days after the massage therapy. Magnetic resonance imaging (MRI) revealed iso-signal intensity on T1-weighted images and high-signal intensity on T2-weighted images of the left distal femur; it also revealed an extraskeletal mass (Fig. 2). Furthermore, MRI revealed diffuse signal alteration in the muscles and the neurovascular areas surrounding the lesion in the distal femur; hence, hemorrhage and dissemination of the tumor were considered (Fig. 2). Consistent with osteosarcoma, open biopsy followed by histopathological examination revealed tumor cell proliferation with extensive pleomorphism and mitoses (Fig. 3). Seventeen days after the massage therapy, computed tomography revealed multiple metastatic lesions in the lung and liver (Fig. 4). Thoracic MRI revealed multiple metastases in the thoracic spine (Fig. 5). ^18^Fluorine-labeled fluorodeoxyglucose-positron emission tomography revealed tumor metastasis in the femur and multiple metastases in the thoracic and lumbar spine, liver, and pelvis (Fig. 6). The patient underwent chemotherapy comprising ifosfamide, carboplatin, pirarubicin, etoposide, doxorubicin, and methotrexate (Fig. 7). During the second course of chemotherapy, paraplegia due to spinal metastases developed and progressed. After eight courses of chemotherapy, the metastatic lesions in the lung and liver reduced in size (Fig. 8), although considerable primary tumor growth was observed (Fig. 9). Subsequently, the patient received hip disarticulation 6 months after the initial diagnosis, and he then underwent radiation therapy for metastatic lesions in the liver and sacrum. However, metastatic lesion growth was observed, and the patient died due to multiple metastases 13 months after the initial diagnosis.

## Discussion and conclusions

Despite the weak evidence regarding its efficacy, massage therapy is widely used to mitigate various types of chronic pain symptoms and to promote return to normal function [14–19]. Indeed, a randomized trial showed that therapeutic massage provides relief from intense pain, improves mood status, and offers muscle relaxation in patients with metastatic bone pain [20]. The possibility that direct compression of a tumor may induce metastasis and dissemination has been considered, although there is little evidence. Therefore, compression due to Esmarch’s bandages and tourniquets are contraindications for tumors in the extremities [21, 22]. Hayashi et al. investigated the association of tumor compression and lymph node metastasis in a mouse model of fibrosarcoma [23]; in vivo fluorescence imaging of the fibrosarcoma cells labeled with a fluorescent protein showed that pressure-dependent compression of the tumor tissue increased the number of tumor cells that shed into the lymph duct. An in vivo study using GFP-labeled osteosarcoma cells demonstrated that massage increases tumor volume as well as metastases in the lymph node and lung [13].

In a retrospective study conducted in Taiwan, 70 of 134 patients (52%) with osteosarcoma underwent alternative medical treatment including massage therapy before their initial visit to the hospital [12]. A remarkable difference was observed in the 5-year overall survival rate–58% in patients treated with massage therapy versus 92% in those not treated massage therapy. However, these results were confounded because prior to the hospital visit, there was a significantly higher incidence of metastatic lung lesions upon initial diagnosis (51% in the massage group vs 19% in the non-massage group) and higher rate of tumor recurrence (29% in the massage group vs 6% in the non-massage group). Another retrospective study showed that massage therapy decreased overall survival and increased incidence of local recurrence and metastases [13]. Thus, due to the fragility of the tumor tissue compared with normal tissue, compression during massage is thought to destroy tissues and rupture tumor vessels. Dissemination of tumor tissue due to hemorrhage renders it difficult to perform limb salvage surgery, thereby impacting survival. In the present case, the association of massage therapy with the dissemination of osteosarcoma cannot be determined because lack of MRI before massage therapy. However, the diffuse signal alteration in the muscles and the neurovascular areas surrounding the tumor observed by MRI is consistent with a cause of the severe pain after massage therapy. Therefore, the present case suggests the local dissemination of tumor tissue due to compression of the osteosarcoma, although the influence of massage therapy on metastatic disease remains unclear. Although massage therapy alleviates several symptoms and brings relief, massage therapists should be aware of the possibility that their massage can disseminate hidden malignancies. In conclusion, the present case suggests the dissemination of tumor tissue due to massage therapy, which while creating awareness regarding this rare but most common malignant bone tumor in youth also cautions massage therapists to be aware of the condition and the outcomes.

## Data Availability

To protect privacy and respect confidentiality, no raw data have been made available in any public repository. The datasets used and/or analyzed during the current study available from the corresponding author on reasonable request.

## Abbreviations

^18^F-FDG: ^18^Fluorine-labelled fluorodeoxyglucose
CT: Computed tomography
GFP: Green fluorescent protein
MRI: Magnetic resonance imaging
PET: Positron emission tomography
